# Supplementary figures and images for: Adipokinetic hormone signaling mediates the enhanced fecundity of Diaphorina citri infected by ‘Candidatus Liberibacter asiaticus’
Source: eLife. 2024 Jul 10;13:RP93450. doi: 10.7554/eLife.93450 (PMC11236419; doi:10.7554/eLife.93450)

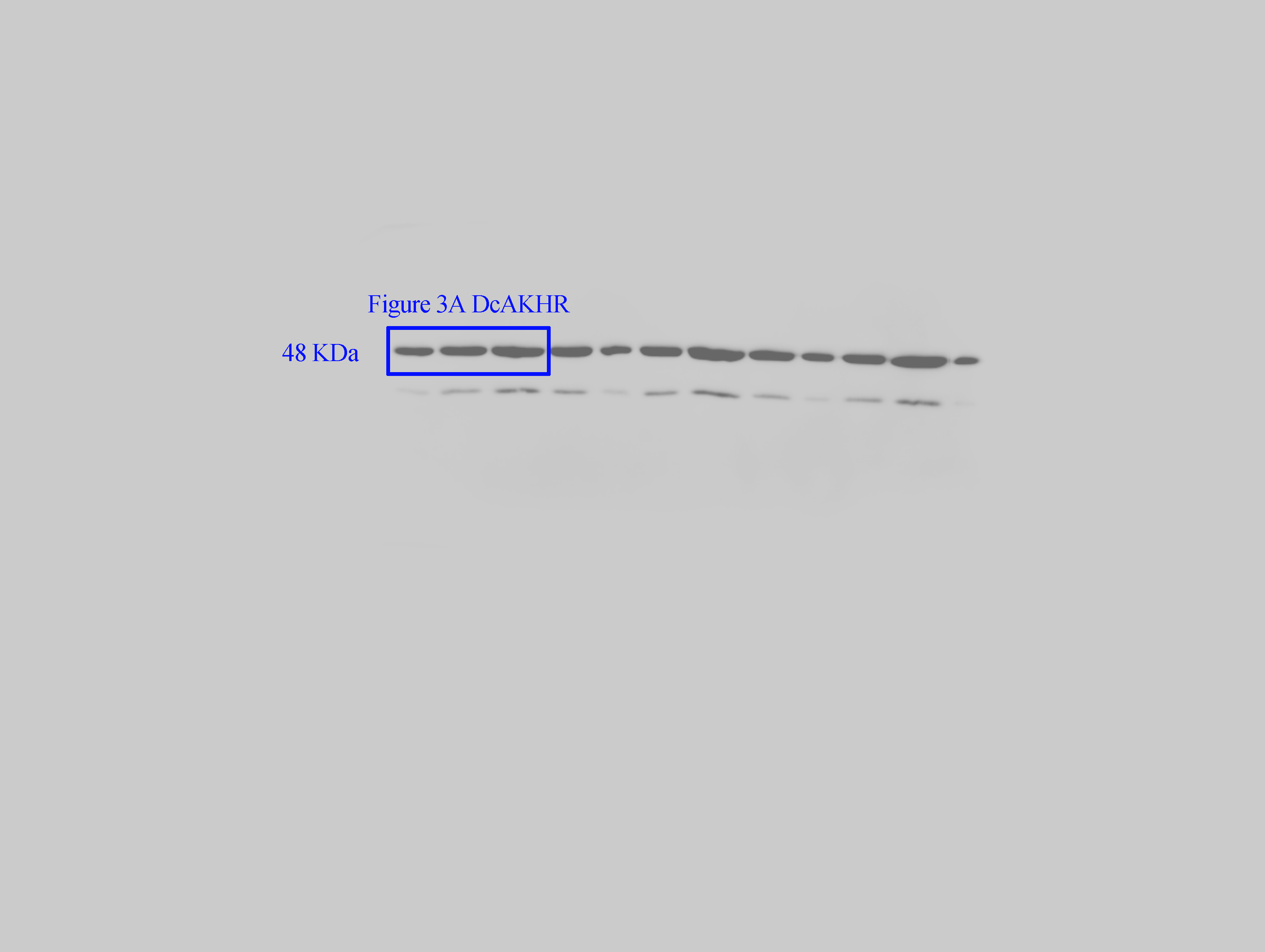

Supplement: Figure 3—source data 1. [file elife-93450-fig3-data1.zip › Figure 3 Source data-1/Figure 3A DcAKHR-labelled.tif]

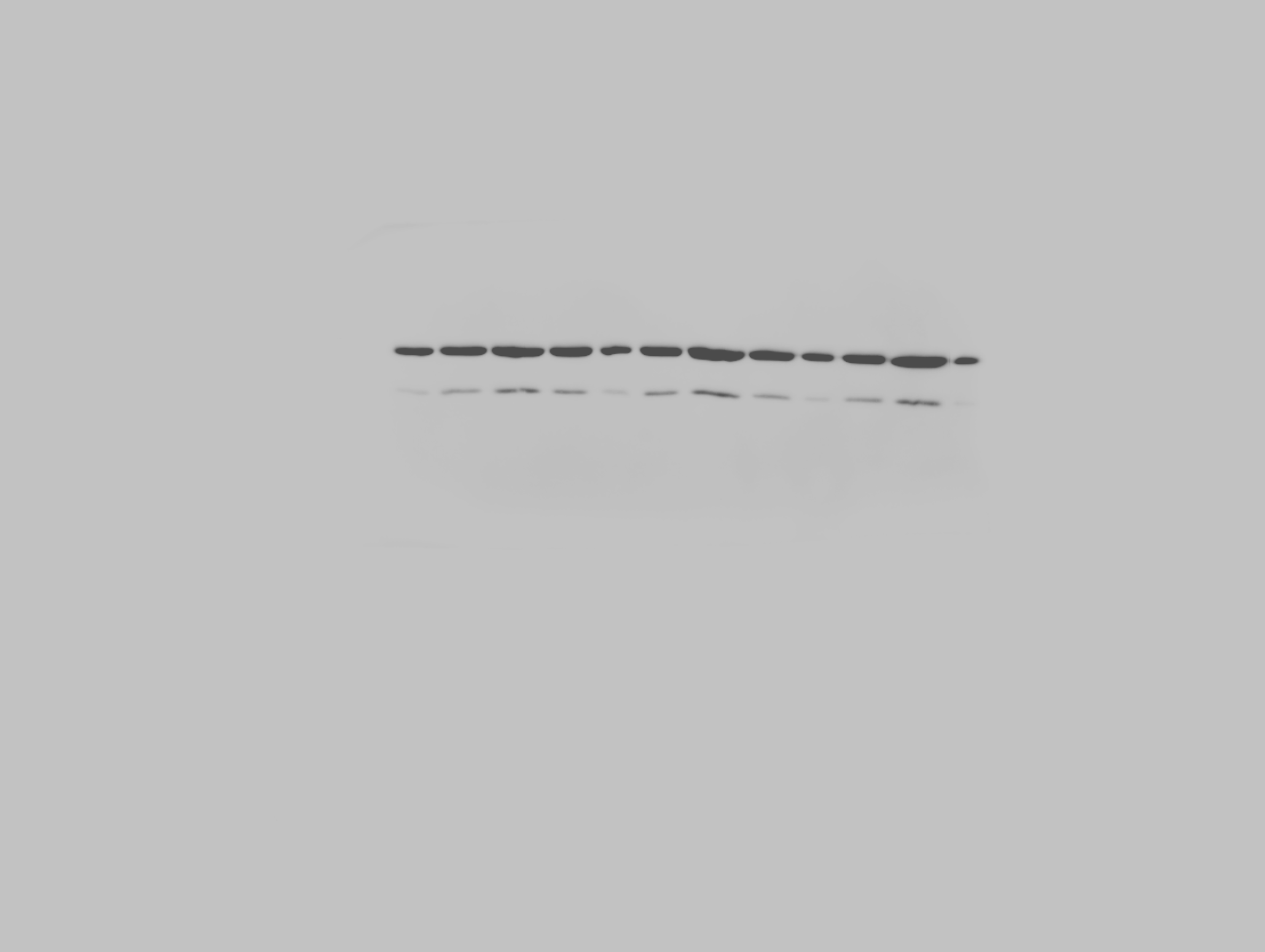

Supplement: Figure 3—source data 1. [file elife-93450-fig3-data1.zip › Figure 3 Source data-1/Figure 3A DcAKHR-original.tif]

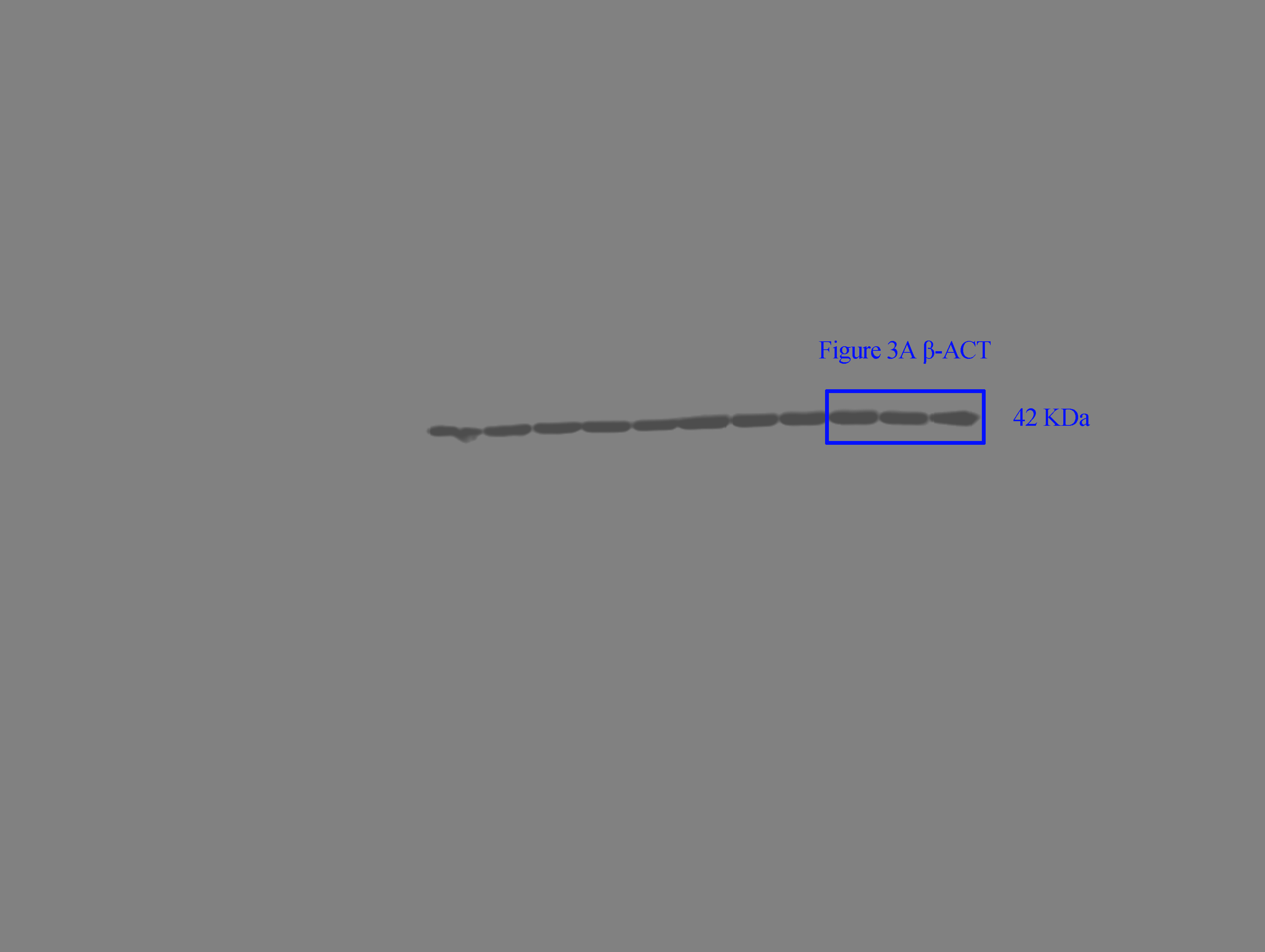

Supplement: Figure 3—source data 1. [file elife-93450-fig3-data1.zip › Figure 3 Source data-1/Figure 3A a┬-ACT-labelled.tif]

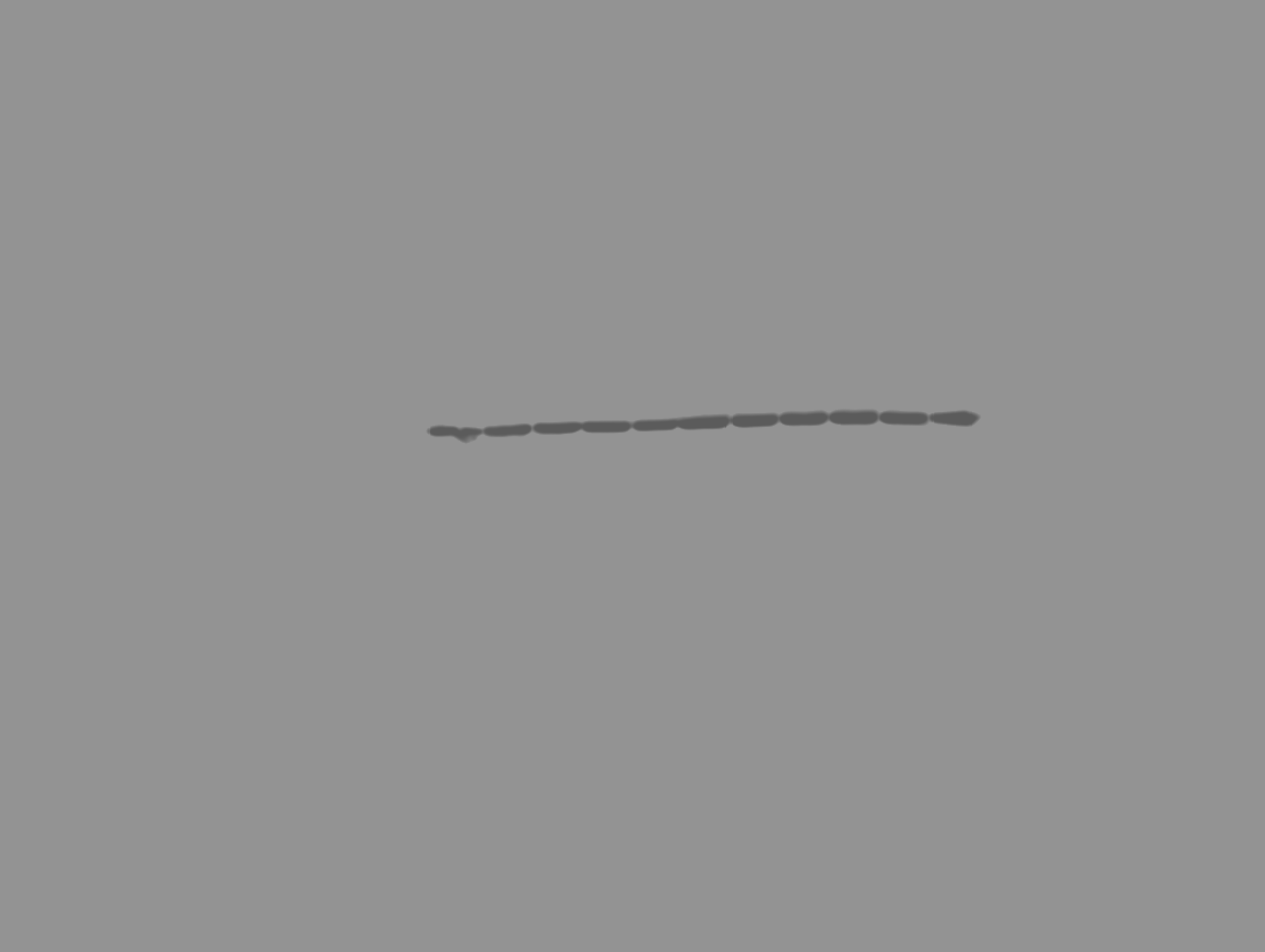

Supplement: Figure 3—source data 1. [file elife-93450-fig3-data1.zip › Figure 3 Source data-1/Figure 3A a┬-ACT-original.tif]

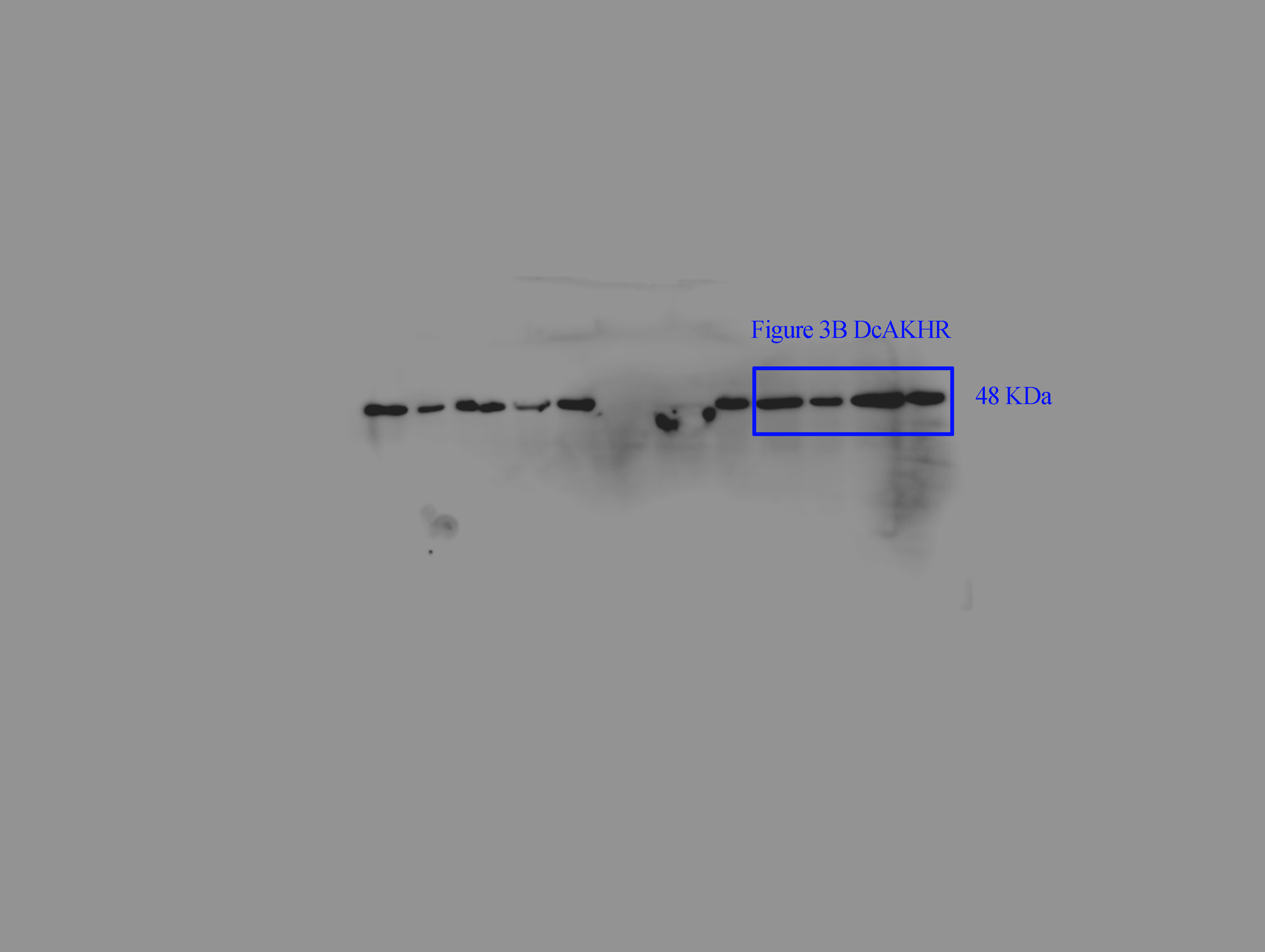

Supplement: Figure 3—source data 1. [file elife-93450-fig3-data1.zip › Figure 3 Source data-1/Figure 3B DcAKHR-labelled.tif]

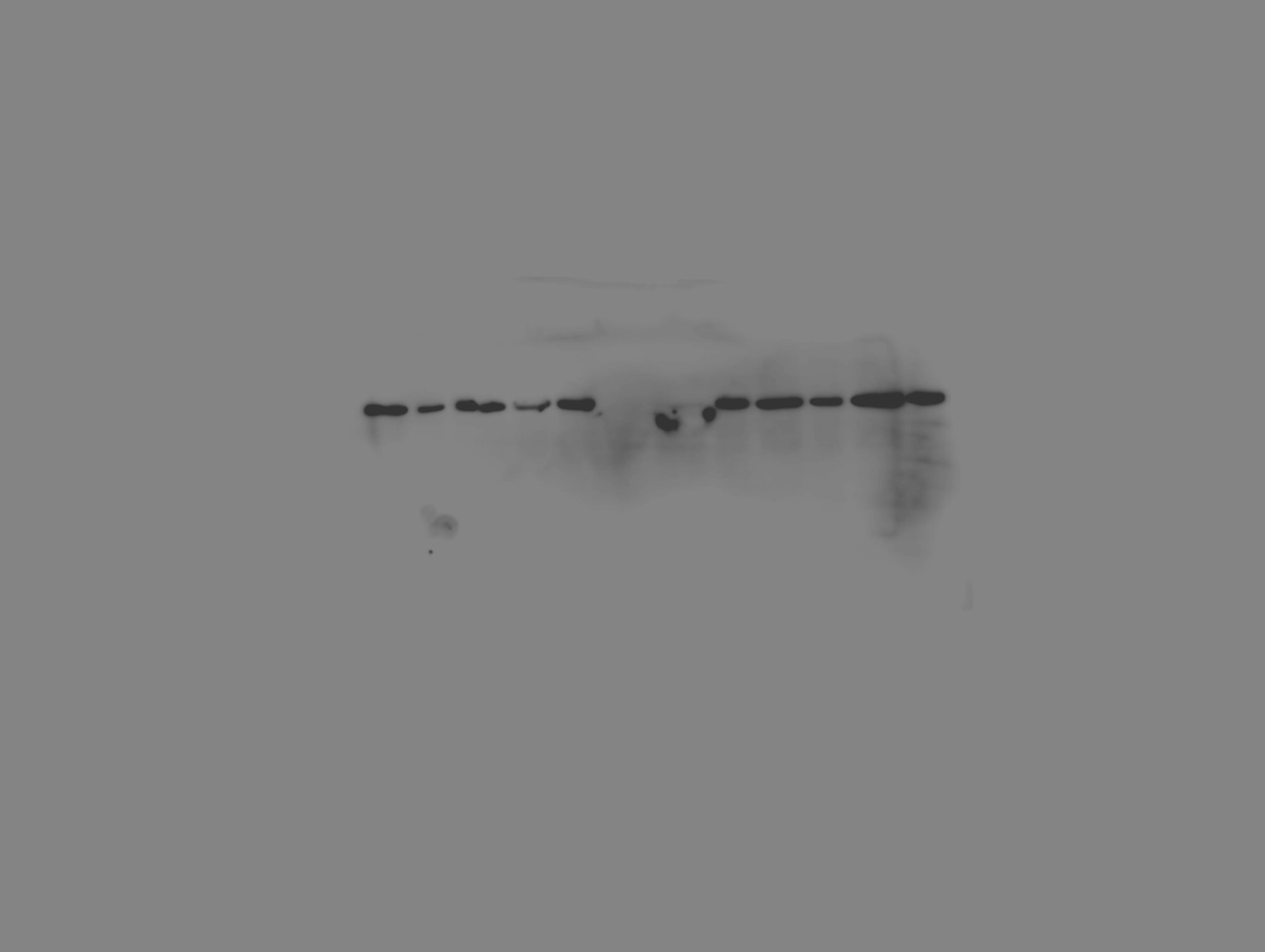

Supplement: Figure 3—source data 1. [file elife-93450-fig3-data1.zip › Figure 3 Source data-1/Figure 3B DcAKHR-original.tif]

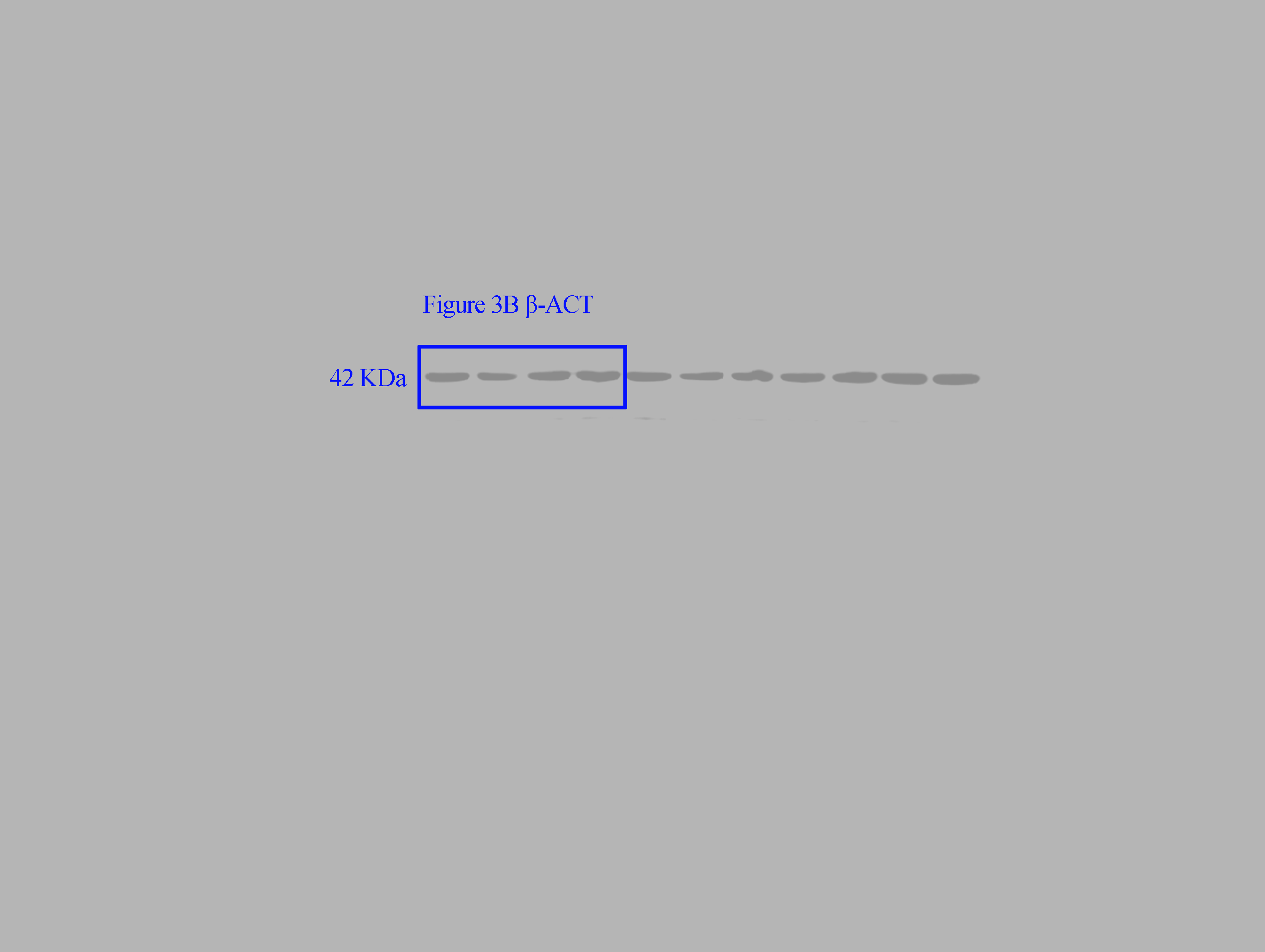

Supplement: Figure 3—source data 1. [file elife-93450-fig3-data1.zip › Figure 3 Source data-1/Figure 3B a┬-ACT-labelled.tif]

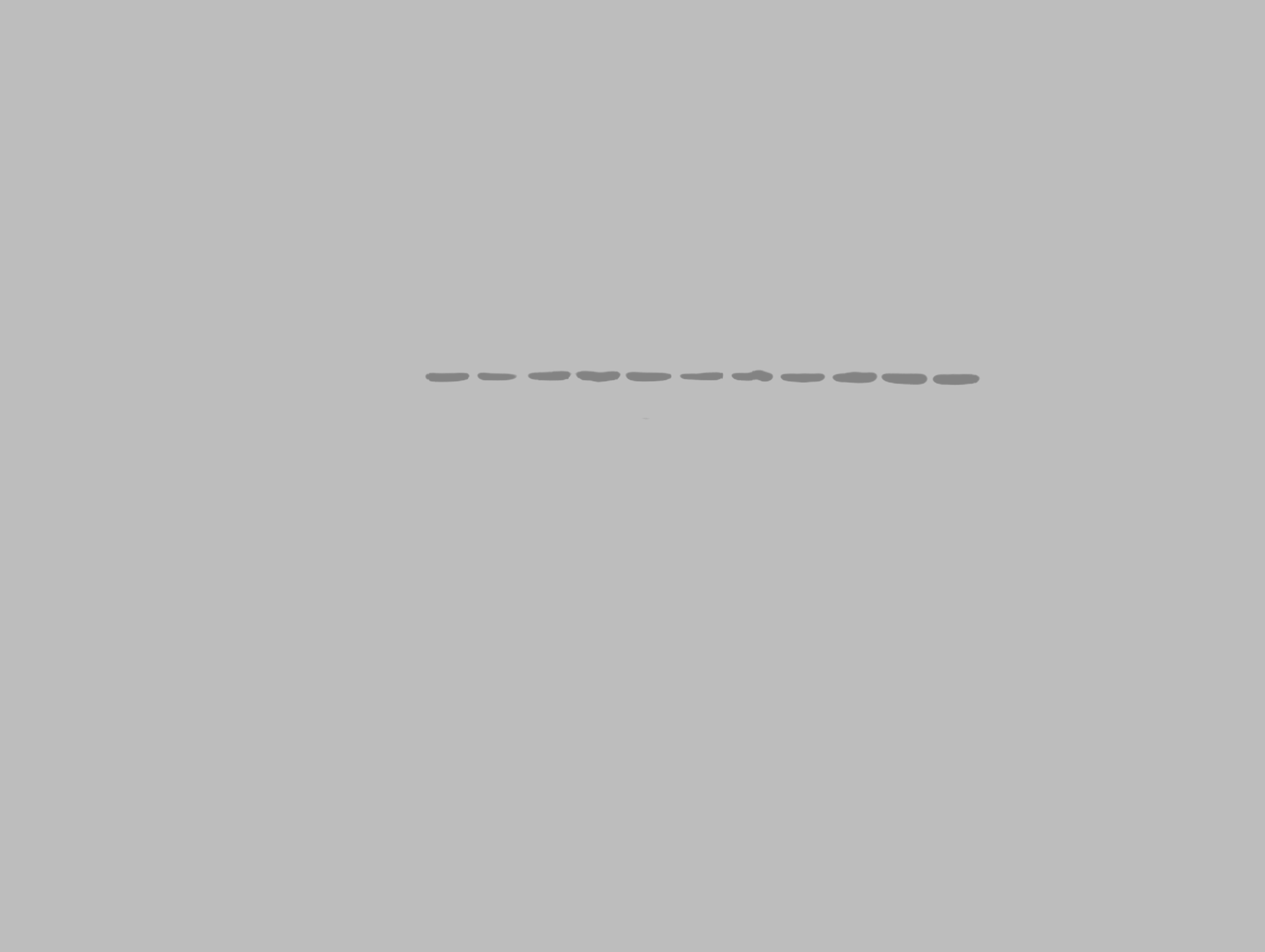

Supplement: Figure 3—source data 1. [file elife-93450-fig3-data1.zip › Figure 3 Source data-1/Figure 3B a┬-ACT-original.tif]

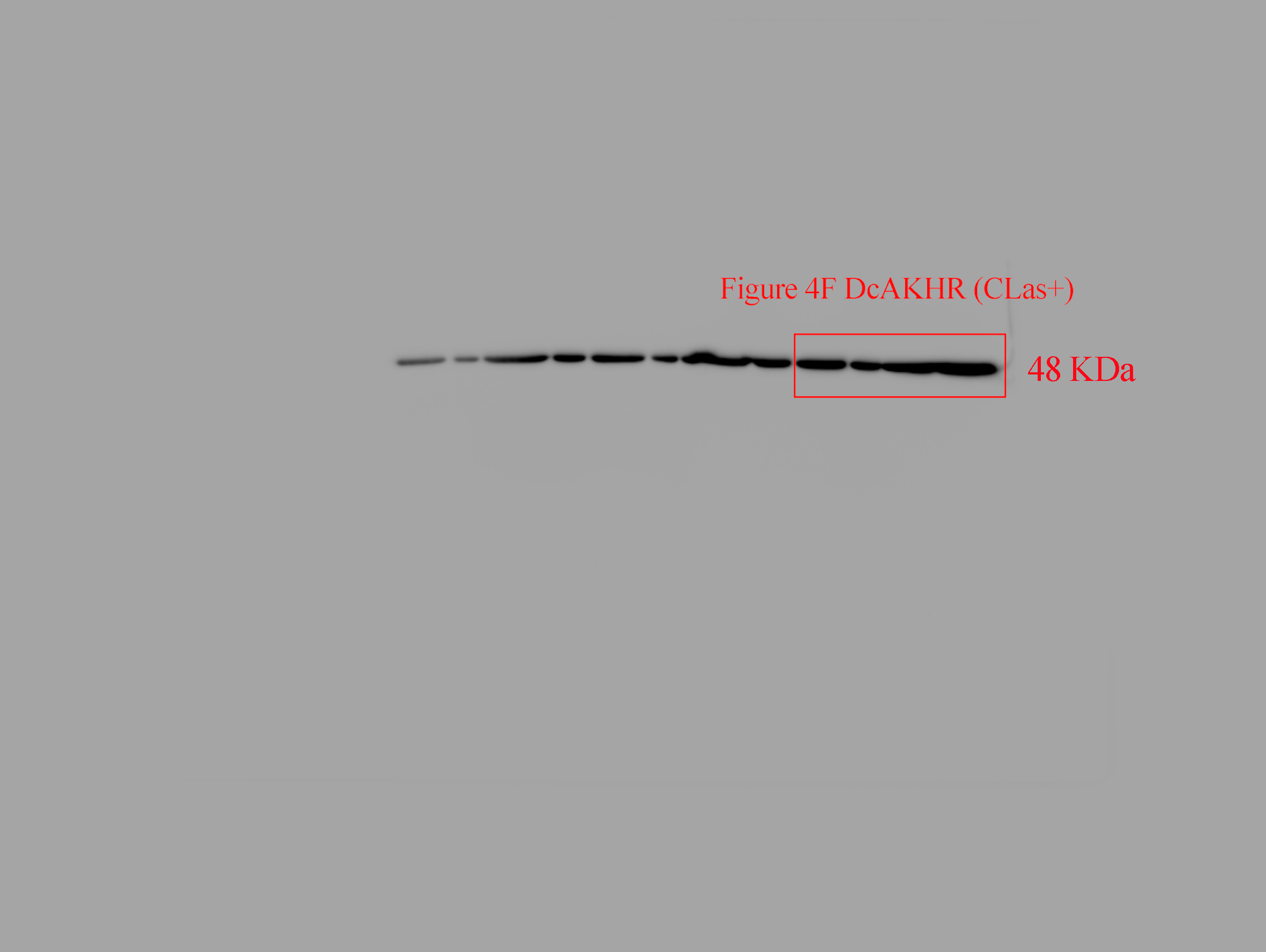

Supplement: Figure 4—source data 1. [file elife-93450-fig4-data1.zip › Figure 4 Source data-1/Figure 4F DcAKHR (CLas+)-labelled.tif]

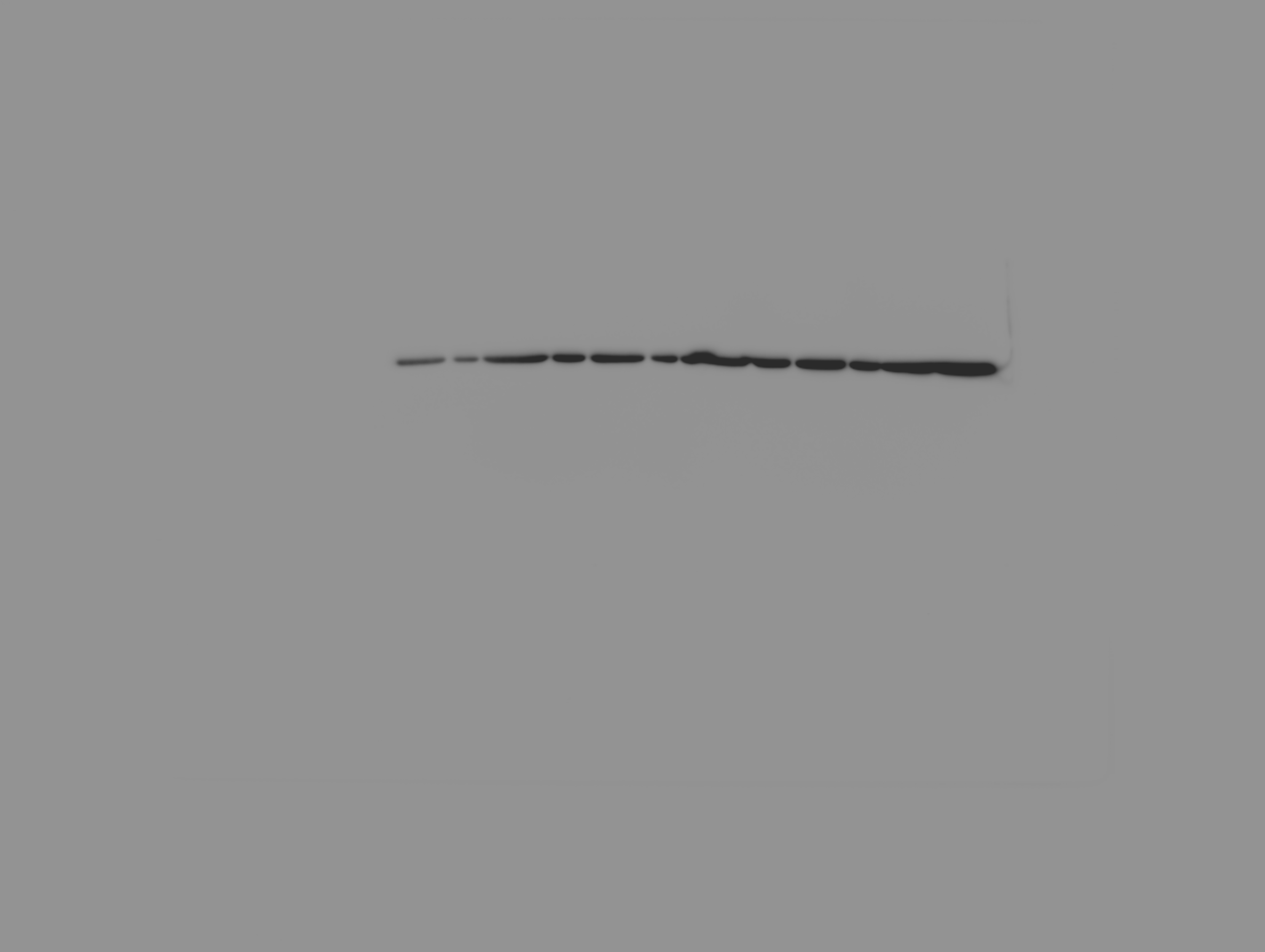

Supplement: Figure 4—source data 1. [file elife-93450-fig4-data1.zip › Figure 4 Source data-1/Figure 4F DcAKHR (CLas+)-original.tif]

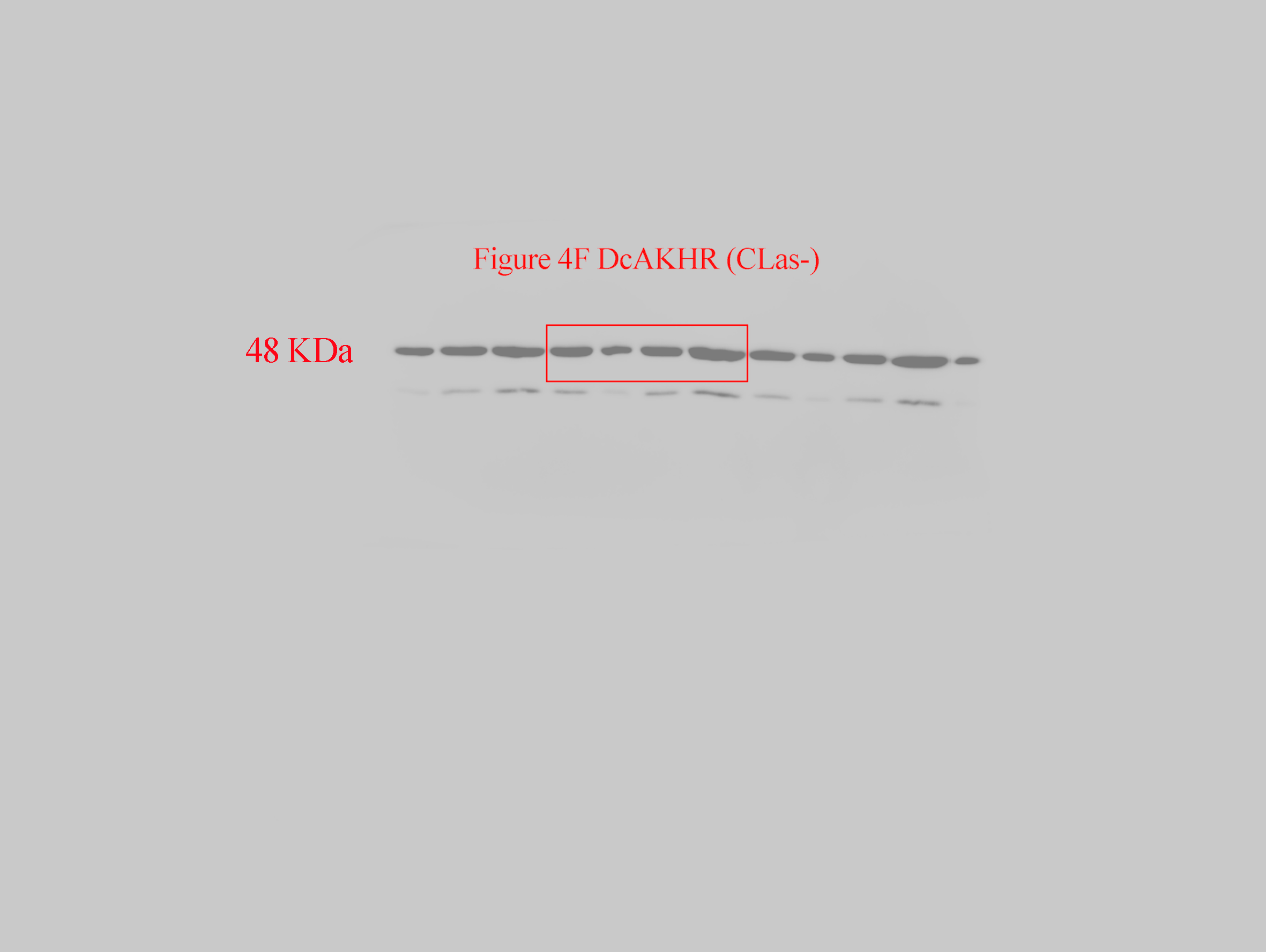

Supplement: Figure 4—source data 1. [file elife-93450-fig4-data1.zip › Figure 4 Source data-1/Figure 4F DcAKHR (CLas-)-labelled.tif]

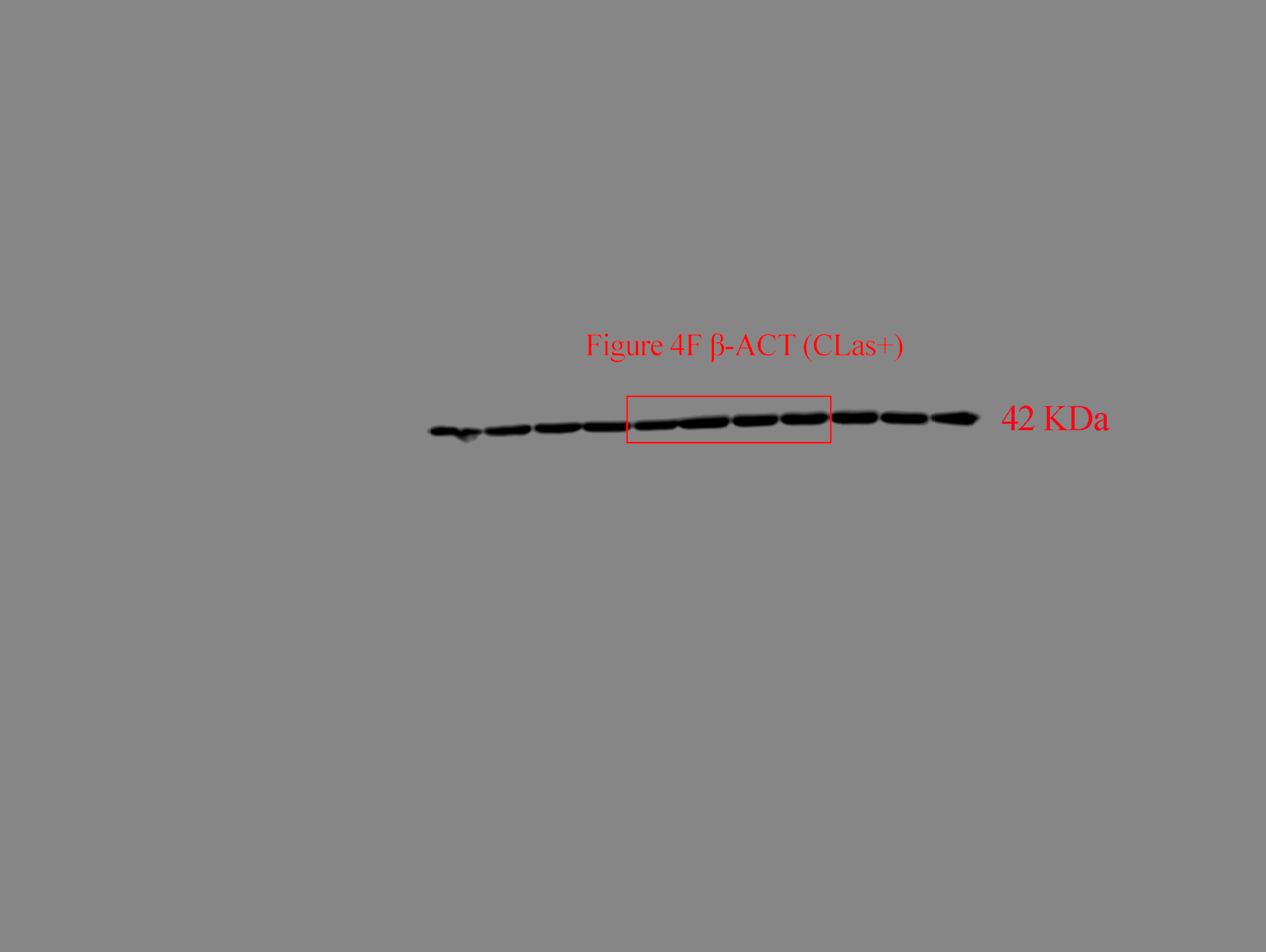

Supplement: Figure 4—source data 1. [file elife-93450-fig4-data1.zip › Figure 4 Source data-1/Figure 4F a┬-ACT (CLas+)-labelled.tif]

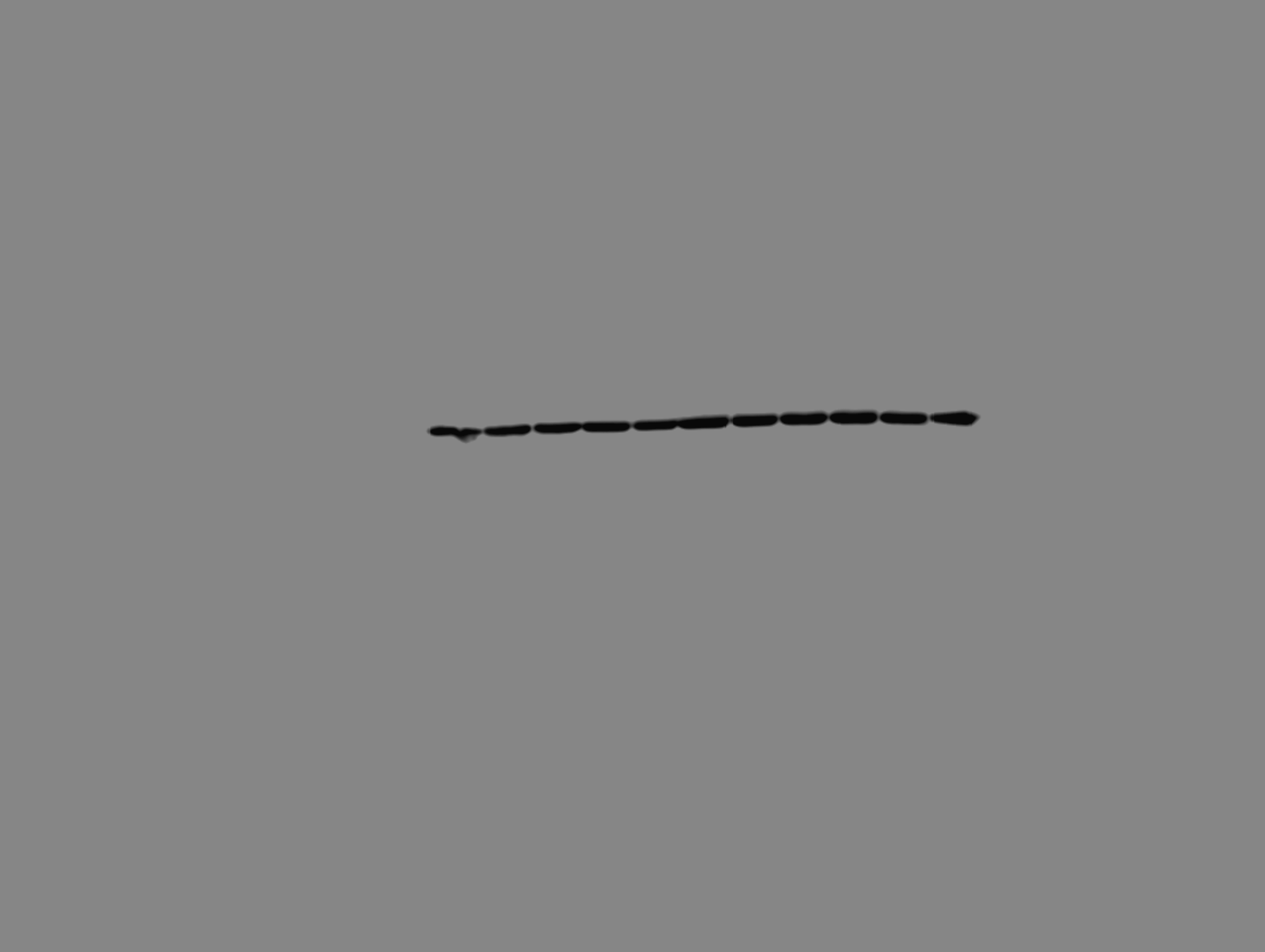

Supplement: Figure 4—source data 1. [file elife-93450-fig4-data1.zip › Figure 4 Source data-1/Figure 4F a┬-ACT (CLas+)-original.tif]

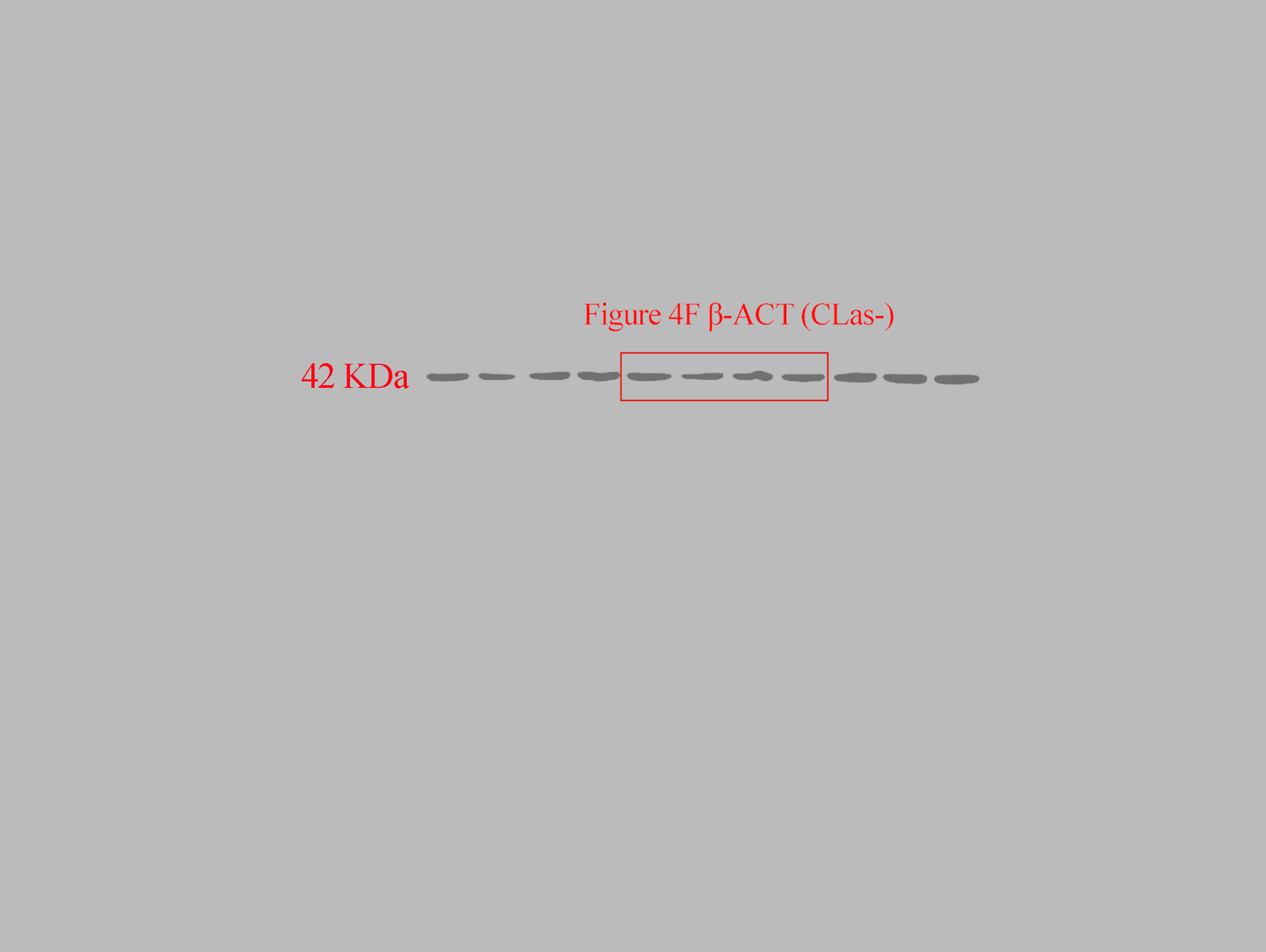

Supplement: Figure 4—source data 1. [file elife-93450-fig4-data1.zip › Figure 4 Source data-1/Figure 4F a┬-ACT (CLas-)-labelled.tif]

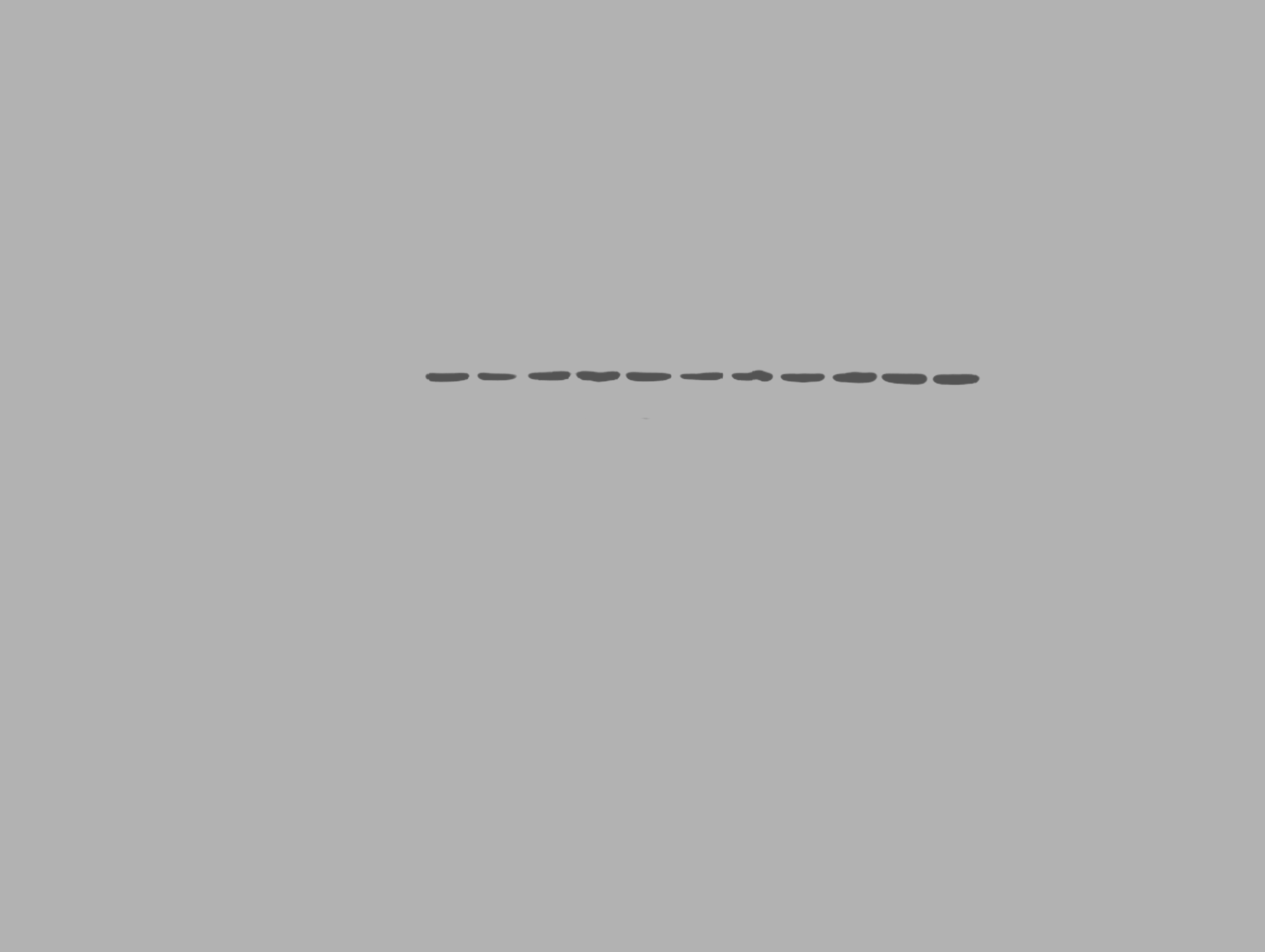

Supplement: Figure 4—source data 1. [file elife-93450-fig4-data1.zip › Figure 4 Source data-1/Figure 4F a┬-ACT (CLas-)-original.tif]
