# Supplementary material for: Adipokinetic hormone signaling mediates the enhanced fecundity of Diaphorina citri infected by ‘Candidatus Liberibacter asiaticus’
Source: eLife. 2024 Jul 10;13:RP93450. doi: 10.7554/eLife.93450 (PMC11236419; doi:10.7554/eLife.93450)
Supplement: Supplementary file 1. — Related to Method details. [file elife-93450-supp1.docx]

**Supplementary file 1**. **Primer list used for 3’RACE, qRT-PCR, and RNAi analysis. Related to Method details.**

| **Gene**  **name** | **Accession number** | **primer name** | **Sequences of primers (5’-3’)** | **Product size (bp)** | **Purpose** |
| --- | --- | --- | --- | --- | --- |
| *DcAKH* | MG550150.1 | *DcAKH*-Full-F | ATGGAGCGACACCATGTGAG | 222 | Validation of full length sequence |
|  |  | *DcAKH*-Full-R | CTGATTGAATTTTTCACATT |  |  |
|  |  | *DcAKH*-qF | TGGACTCTCTCAAGTACATTT | 54 | qRT-PCR |
|  |  | *DcAKH*-qR | ACAATTTTTGGGCTTCACTC |  |  |
|  |  | *DcAKH*-RNAi-F | taatacgactcactatagggATGGAGCGACACCATGTGAG | 138 | dsRNA synthesis |
|  |  | *DcAKH*-RNAi-R | taatacgactcactatagggGCGACATTCATCAGGAGGAT |  |  |
|  |  | *DcAKH* mature peptide | pQVNFSPNW-NH2 | - | mature peptide |
|  |  | *DcCrZ* mature peptide | pQTFQYSRGWTN-NH2 | - |  |
| *DcAKHR* | OR259432 | *DcAKHR*-Full-F | CACATCACCCGGTATGATT | 1452 | Validation of full length sequence |
|  |  | *DcAKHR*-Full-R | CTCCCCAAGATTCAACCTT |  |  |
|  |  | *DcAKHR*-3UTR-F | CTGATACGGCCAATAACGTG | 185 | 3’UTR amplification |
|  |  | 3’RACE-Outer Primer | TACCGTCGTTCCACTAGTGATTT |  |  |
|  |  | *DcAKHR*-qF | TCAGTTGGAAGAAGGACAAG | 116 | qRT-PCR |
|  |  | *DcAKHR*-qR | GGTACTCAGGCTACACTCTA |  |  |
|  |  | *DcAKHR*-RNAi-F | taatacgactcactatagggCTGAGTGGCCTCTGCCTAAC | 399 | dsRNA synthesis |
|  |  | *DcAKHR*-RNAi-R | taatacgactcactatagggTTAACGGCTTCAAAACTGCC |  |  |
|  |  | *DcAKHR*-3UTR-Full-F | CTAGTTGTTTAAACGAGCTCTAAGAGAAGAGACTGGCCGG | 166 | Full sequence of 3’UTR |
|  |  | *DcAKHR*-3UTR-Full-R | TGCATGCCTGCAGGTCGACTCTAGACACAGGAGTTTCAATCTATT |  |  |
|  |  | *DcAKHR*-3UTR-mutant-F | CTAGTTGTTTAAACGAGCTCGCCACATTGCGTTAAACCGT | 166 | Mutant sequence of 3’UTR |
|  |  | *DcAKHR*-3UTR-mutant-R | TGCATGCCTGCAGGTCGACTCTAGACACAGGAGTTTCAATCTATT |  |  |
|  |  | *DcAKHR*-probe | FAM-CACGTTATTGGCCGTATCAG | - | Labled with FAM for FISH |
| *GFP* | ACY56286 | GFP-RNAi-F | taatacgactcactatagggACTCCAGCAGGACCATGTGATC | 596 | dsRNA synthesis |
|  |  | GFP-RNAi-R | taatacgactcactatagggACCTGAAGTTCATCTGCACCAC |  |  |
| *Dcβ-ACT* | DQ675553.1 | β-ACT-qF | TGTTCCAACCTTCCTTCCTG | 109 | qRT-PCR |
|  |  | β-ACT-qR | GTGTTGGCGTACAGGTCCTT |  |  |
| *DcMet* | OP251123 | *DcMet*-qF | AAGCTCAAGGGCCAAGTCAT | 257 | qRT-PCR |
|  |  | *DcMet*-qR | TAATCAATACCAGGGGCGGC |  |  |
| *DcKr-h1* | XM_026820026.1 | *DcKr-h1-qF* | CTCCAGTGCTGAGTCCACAA | 103 | qRT-PCR |
|  |  | *DcKr-h1-qR* | ATCTCCCGGAGGTTTCTGTT |  |  |
| *DcVg-1-like* | XM_008488883.3 | *DcVg-1-like-qF* | CACCTACTCCTTGTCCTCTA | 166 | qRT-PCR |
|  |  | *DcVg-1-like-qR* | GAAAAATCCCCAGAGTCCTT |  |  |
| *DcVg-A1-like* | XM_026832896.1 | *DcVg-A1-like-qF* | CTCCTCAGAAAGTGGAAGTT | 132 | qRT-PCR |
|  |  | *DcVg-A1-like-qR* | TTGTTTCCGATGAAGTAGGG |  |  |
| *DcVgR* | OP251122 | *DcVgR-qF* | AGCAGCTGGATATACATGTG | 186 | qRT-PCR |
|  |  | *DcVgR-qR* | CTCCACAGTACTGATTACCG |  |  |
| *C*Las 16s rRNA | L22532.1 | *C*Las 16s-probe | Cy3-CATTATCTTCTCCGGCG | - | Labled with Cy3 for FISH |
|  |  | *C*Las 16s-qF | TCGAGCGCGTATGCAATACG | - | qRT-PCR |
|  |  | *C*Las 16s-qR | GCGTTATCCCGTAGAAAAAGGTAG |  |  |
| miR-34 | - | miR34-qF | TGGCAGTGTGGTTAGCTGGTTG |  | qRT-PCR |
|  |  | miR-34-probe | Cy3-CACAACCAGCTAACCACACTGCCA | - | Labled with Cy3 for FISH |
| U6 | - | U6-qF | AGGATGACACGCAAAATCGT | - | qRT-PCR |

Note: The lowercase with underline indicated the T7 promoter sequences in the primers of dsRNA synthesis. The sequences with black boxes displayed the restriction enzyme cutting sites.
